# Identification of differentially expressed genes and fusion genes associated with malignant progression of spinal cord gliomas by transcriptome analysis

**DOI:** 10.1038/s41598-019-50072-9

**Published:** 2019-09-19

**Authors:** Dong-kang Liu, Jin Wang, Yi Guo, Zhen-xing Sun, Gui-huai Wang

**Affiliations:** 1 Department of neurosurgery, Beijing Tsinghua Changgung Hospital, Beijing, 102218 China; 2 School of Clinical Medicine, Tsinghua University, Beijing, 100084 China

**Keywords:** Cancer genomics, CNS cancer

## Abstract

Glioma, the most common histological subtype of primary spinal cord tumors, is considered as a rare central nervous system neoplasm. In this study, 9 glioma samples (4 of grade II and 5 of grade IV with H3K27M positive) were analyzed to examine the molecular mechanisms underlying the malignant progression of gliomas, transcriptome sequencing. Differentially expressed genes (DEGs) in grade IV vs. grade II were analyzed by using the Limma package in R. Enrichment analysis was performed for the individual DEGs through VennPlex software and the Database for Annotation. Gene mutations and fusions were analyzed using the Genome Analysis Toolkit and STAR-Fusion. A total of 416 DEGs were identified in grade IV vs. grade II. Functional analysis of the DEGs showed that *GALR1* and *GRM5* of neuroactive ligand-receptor interactions signaling pathways may be relaed to malignant progression of gliomas. Further systematic transcriptional profiling identified 11 in-frame/frameshift gene fusions in the tumors. Notably, one novel gene fusions, *GATSL2-GTF2I was* detected in all of the grade II samples. In summary, the molecular alterations observed in glioma progression may improve the characterization of different human spinal cord glioma grades. The transcriptome analysis of intramedullary spinal cord glioma will provide a new candidate gene list for further mechanism research.

## Introduction

Intramedullary spinal cord gliomas account for about 2 to 4% of central nervous system (CNS) tumors^1–3^. Of these spinal gliomas, astrocytomas is the most common subtype. Spinal cord gliomas is classified from grade I (least aggressive) to grade IV (most aggressive) according to conventional histopathological criteria based on the World Health Organization (WHO) grading system^4^. This grading system was dramatically revised in 2016,and the the genetic profiles of tumors^5^ was added into it. As one of the deadliest human cancers, grade IV glioma, also termed as glioblastoma (GBM), has a poor median survival time of 12 to 18 months^6,7^. Although the majority of GBMs are primary tumors, around 20% of gliomas still progress from grade II or III^4^. Therefore, the identification of early clinical biomarkers for glioma diagnosis and prognosis is urgently required in clinical practice.

Molecular variations in *IDH1*, *IDH2*, *H3F3A*, *HIST1H3B*, and *BRAF* have been frequently identified in different grades of brain gliomas. However, there are very few researches concerning the molecular indicators of spinal gliomas. High-throughout transcriptome sequencing is capable of capturing the transcriptomic landscape of tumors, including protein-coding and non-coding gene expression, the identification of non-coding RNAs (ncRNAs) and/or fusion genes, and the determination of gene mutations and/or alternative splicing^8,9^. The detection of molecular variants across the each grades of spinal gliomas at the transcriptional level will provide further evidence of the mechanisms in the development and progression of spinal glioma.

In this study, transcriptome sequencing of 9 spinal cord glioma samples were performed. The samples included grade II (control) and grade IV tissues. DEGs and gene fusions were identified in different grades of spinal gliomas samples including tissues of grade II (control) and grade IV.he samples included grade II (control) and grade IV tissues. DEGs and gene fusions were identified in different grades of spinal gliomas samples including tissues of grade II (control) and grade IV. 416 DEGs in IVvs II were identified in this study. Functional analysis suggested that the DEGs are involved in canonical GBM signaling and other relevant signaling pathways. What’s more, 11 in-frame gene fusions were identified according to further systematic transcriptional profiling.

## Results

### Clinico-pathological characteristics of patients

The clinico-pathological characteristics of the 9 patients in our transcriptome analysis are summarized in Table 1. The median age was 24.0 years old (range from 3 to 56 years old) and 55.56% of the patients are male. Of these 9 patients, 4 was in grade II, 5 was in grade IV with H3K27M positive, and 66.67% of them had recurrence after surgery. Except for H3K27M, other genes including BRAF (V600E), ATRX (all mutations that may affect protein function), TERT (C228T, C250T), IDH1 (R132), IDH2 (R172) mutation were analysed (Table 2). ATRX mutation (C2671G) were detected in both Grade II (S01 and S04) and grade IV (S06 and S07).

**Table 1:** Patient Characteristics.

Characteristics	All patients (N = 9)	
	No.	%
Age classification		
<18	3	33.33
18–49	4	44.45
≥50	2	22.22
gender		
Male	5	55.56
Female	4	44.45
WHO grade classes		
II	4	44.45
IV	5	55.55
Postoperative information		
Normal	2	22.22
Recurrence	6	66.67
Lost	1	11.11

**Table 2:** Mutations of the gene identified in each sample.

Sample ID	Gene list					
	H3(K27M)	BRAF (V600E)	ATRX	TERT (C228T, C250T)	IDH1 (R132)	IDH2 (R172)
S01 (grade II)	—	—	C2671G	—	—	—
S02 (grade II)	—	—	—	—	—	—
S03 (grade II)	—	—	—	—	—	—
S04 (grade II)	—	—	C2671G	—	—	—
S05 (grade IV)	K27M	—	—	—	—	—
S06 (grade IV)	K27M	—	C2671G	—	—	—
S07 (grade IV)	K27M	—	C2671G	—	—	—
S08 (grade IV)	K27M	—	—	—	—	—
S09 (grade IV)	K27M	—	—	—	—	—

### Identification of DEGs

To gain insight into the molecular pathogenesis of spinal gliomas among the Chinese population, we searched for genetic alterations in 9 spinal gliomas by transcriptome analysis.

RNA-Seq analysis included 264 million reads. An average of 29 million short-sequence reads was obtained for each individual sample and more than 81.05% of total reads were identified as clean reads (Supplementary Table S1).

The total clean reads were then mapped to the human whole-genome assembly. The expression level of each gene was normalized and evaluated by the FPKM approach. By comparing the expression of different grades of spinal gliomas, significance differential expression of 416 DEGs were identified in grades IV vs II (Supplementary Table S1).

### Identification of neuroactive ligand-receptor interaction signaling pathways

The most enriched pathways between the astrocytoma grades were identified. A total of 198 differential pathways were identified in IV vs II, including Neuroactive ligand-receptor interaction (11 genes), Cellular senescence (8 genes), cGMP-PKG signaling pathway (7 genes) and so on (See Supplementary Table S3). The gene lists of the Neuroactive ligand-receptor interaction, Cellular senescence, and MAPK signaling pathway were shown in Table 3.

**Table 3:** DEGs involved in neuroactive ligand-receptor interaction signaling pathways associated with spinal cord gliomas progression.

Pathway	Gene symbol	Log2 (IV vs II)	*P* value	FDR-adjusted *P*-Value
Neuroactive ligand-receptor interaction	*NTS*	−7.36	5.96609E-11	1.26E-07
	*NPY5R*	−3.66	4.31307E-07	0.000228688
	*GRM5*	4.10	6.13001E-07	0.000296166
	*NPY2R*	−6.93	2.50691E-06	0.000862973
	*NMU*	−4.88	3.05069E-06	0.000987559
	*GALR1*	−2.99	4.62803E-06	0.001341594
	*NPY1R*	−2.12	1.00139E-05	0.002317425
	*AGTR1*	−5.17	1.18406E-05	0.00267278
	*GNRH1*	−1.89	0.000138916	0.016006712
	*GABRE*	−2.49	0.000194767	0.020394237
	*GAL*	−4.19	0.000268375	0.025345406
Cellular senescence	*SERPINE1*	−2.84	1.96211E-05	0.003859601
	*HLA-F*	−4.88	8.68899E-05	0.011639023
	*GADD45A*	−2.38	0.000105753	0.013359327
	*ITPR2*	1.57	0.000107934	0.013552805
	*CCNA1*	3.06	0.000425885	0.035010305
	*IGFBP3*	−3.16	0.000438499	0.035309435
	*HLA-A*	−5.58	0.000541247	0.040725151
	*SLC25A5*	1.52	0.000604647	0.043819385
MAPK signaling pathway	*NPPA*	−5.12	5.13175E-09	6.14E-06
	*AGTR1*	−5.17	1.18406E-05	0.00267278
	*ITPR2*	1.57	0.000107934	0.013552805
	*ATP2B3*	3.41	0.000142409	0.016273075
	*CNGB1*	−6.15	0.000169499	0.01837725
	*ATP1A2*	2.59	0.000252986	0.024531604
	*SLC25A5*	1.52	0.000604647	0.043819385

Note:

Log_2_ FoldChange (IV vs II): The multiple value of difference between two samples or comparison combinations, expressed as log_2_ (group1/group2), calculated according to the difference analysis software.

*P* value: *P* value of significance test.

FDR-adjusted *P*-Value: *P* value of multiple hypothesis test correction.

Functional annotations of the DEGs in IV vs II showed that Neuroactive ligand-receptor interaction accumulated the highest number of dysregulated genes, which suggested the close association with spinal glioma progression (Table 3). among the 11 genes, *GRM5, GALR1, NPY1R and AGTR1* has been reported playing a role in tumors’ development and progression. Therefore, these candidate genes could be used for future research.

### Detection of gene fusions

Gene fusions can be identified by searching paired-end reads for two ends mapping to different genes, or for reads containing sequences from two different genes. Of the 44 fusion transcripts from spinal glioma tissues, 11 were in-frame/frameshift and 33 were out of frame (Table 4). Of the in-frame fusions/frameshift, 9 originated from the fusion of sequences located on the same chromosome,and 2 arose from sequences derived from different chromosomes. The majority of gene fusions were from chromosome 1, 7 and 17. 6 out of 11 (54.5%) fusion genes were confirmed by real-time PCR.

**Table 4:** Fusion genes identified in grade II and grade IV spinal cord gliomas.

Sample ID	Fusion Gene	LeftBreakpoint	RightBreakpoint	Verified By RT-PCR
S01(Grade II)	RNF213-SLC26A11	chr17:78324196	chr17:78221929	No
S01/S02/S03/S04(Grade II)	GATSL2-GTF2I	chr7:74867229	chr7:74143124	Yes
S03(Grade II)	KIAA1549-BRAF	chr7:138552721	chr7:140487384	Yes
S06(Grade IV)	RC3H1-PKLR	chr1:173947626	chr1:155260469	No
S06(Grade IV)	MEX3B-KIAA1199	chr15:82337791	chr15:81212435	Yes
S08(Grade IV)	ABL2-NCF2	chr1:179095512	chr1:183529408	Yes
S09(Grade IV)	CASK-SUGP1	chrX:41646431	chr19:19427402	No
S09(Grade IV)	TMEM165-PDGFRA	chr4:56262563	chr4:55133456	No
S09(Grade IV)	SLC35F1-ECHDC1	chr6:118588317	chr6:127611422	Yes
S09(Grade IV)	TCF12-SCFD2	chr15:57213296	chr4:54140168	Yes
S06(Grade IV)	CPD-SEZ6	chr17:28712254	chr17:27284557	No

Note:

Left Break point: The genome position where the upstream gene break point is located.

Right Break point: The genome position where the downstream genes break points is located.

Verified By RT-PCR**:** “Yes” mean that the fusion genes were verified by RT-PCR sucessfully, and “No” mean that the fusion genes were not verified by RT-PCR successfully.

Of the 11 fusion genes, *GATSL2-GTF2I*, were detected in all of four gradeII samples. Other 10 fusion genes were only detected in one sample of grade II or grade IV.

## Discussion

In this study, molecular alterations of spinal gliomas were reported by transcriptome analysis, including changes in gene expression, gene fusions, and the mutational landscape of grade II and IV tumors. Totally, 74 DEGs were identified in IV vs II. The identification of the enriched pathways of the DEGs provides information about the cellular processes, which was affected from one spinal glioma grade to the other. Therefore, the most enriched pathways across the astrocytoma grades were compared comprehensively. A total of 187 differential pathways were identified in IV vs II, including neuroactive ligand-receptor interactions (5 genes), hippo signaling (2 genes), Glycosaminoglycan biosynthesis and degradation (10 genes), and pathways in apoptosis (2 genes). These quantitative results suggest that key signaling pathways become increasingly related with the dysregulation of spinal glioma progression. Functional annotations (Supplementary Table S2) of the DEGs in IV vs II showed that neuroactive ligand-receptor interaction pathways accumulated the highest number of dysregulated genes, suggesting their association with spinal glioma progression (Table 3). A total of 2 genes (*GRM5/GALR1*) with known roles in neuroactive ligand-receptor interactions were detected in IV vs II. These quantitative results further support the notion that these pathways become increasingly dysregulated with the progression of glioma malignancy. Neuroactive ligand-receptor signaling pathways accumulated the most DEGs in group’s IV vs II, Which suggested the association with spinal glioma progression. Similarily, the enrichment of Neuroactive ligand-receptor interactions was also reported in previous glioma studies^10–12^. Among them, *GALR1* and *GRM5* were further investigated due to their significant dysregulation in IV vs II. Among the relevant candidate genes involved in tumor progression, *GALR1* encodes galanin receptor 1 (GALR1), which is most likely responsible for the GAL binding observed in glioblastomas, idicating its influence on GBM differentiation and growth^13^. What’s more it is also suggested that *GALR1* mutations are responsible for the overexpression of *GALR1* in grade IV tumors. *GRM5* encodes the metabotropic glutamate receptor 5 that mediates post-synaptic NMDA receptor (NMDAR) currents^14^. It was reported that GRM5 was aberrantly expressed in brain gliomas^15^, suggesting its involvement in both brain and spinal glioma progression. The identification of genes associated with spinal gliomas provides new therapeutic targets and facilitates the development of biomakers for early tumor screening.

In our profiling of the 9 spinal glioma samples, 11 in-frame/frameshift fusions were identified. Notably, these identifications were increased with tumor progression from grade II to IV. This prevalence was consistent with the previously reported study in brain gliomas^16^, which means that the proportion of fusions in grades IV are higher than that in grade II. *KIAA1549-BRAF* fusions have been identified^17^ and it was considered a causes to MAPK activation in pilocytic astrocytoma^17–19^. Besides, those previously reported studies, we have identified a number of novel fusions, of which *GATSL2-GTF2I* were detected in all of grade II samples, suggesting that the gene fusions could be related with the abnormal gene expression observed in spinal gliomas. Most of the identified fusion transcripts involved gene sequences have not been studied in GBMs. Therefore, characterizing the function of these fusions may unravel novel biological mechanisms of spinal glioma progression.

In conclusion, a complex landscape of molecular alterations in spinal gliomas across different tumor grades was revealed in this study. This advances our understanding of the progression of these tumors in the Chinese population. Further investigations of the network of these genes will further identify the characterization of their underlying mechanisms during the development of aggressive spinal gliomas.

## Methods

### Patients and samples

Tissues were collected from patients undergoing treatment at the Beijing Tiantan Hospital and Beijing Tsinghua Changgung Hospital from 2012 to 2017. Informed consent was obtained from study participants according to institutional guidelines. Tissue samples were snap-frozen in liquid nitrogen. Tumor grades were diagnosed by two experienced pathologists. The clinical information of each patient is shown in Table 1.

All procedures involving human tumor specimens were performed in accordance with the ethical standards of our local research committee and the Helsinki declaration The study was approved by the Human Research Ethical Committee of Beijing Tsinghua Chenggung Hospital, China.

### Histopathological diagnosis and mutation detection

Each histopathological slide of the patient sample was reviewed by at least one experienced neuropathologist. The H3K27M, BRAF (V600E), ATRX (all mutations that may affect protein function), TERT (C228T, C250T), IDH1 (R132), IDH2 (R172) mutation status were sequenced by Genome Analysis Toolkit (GATK) which can be used to identify H3K27M mutation type.

### RNA-seq and quality control

RNA library construction and sequencing experiments were conducted at Anhui Anlongen Co., Ltd., (Hefei, China). Libraries were sequenced on an Illumina HiSeq 2000 platform using 150-bp paired-end sequencing. Generated images were converted into nucleotide sequences using a base-calling pipeline. Raw reads were saved in the fastq format and subjected to standard quality control (QC) criteria to remove the unfitted according to the following parameters: (1) reads aligned to adaptors or primers; (2) reads with >10% unknown bases (N bases); (3) reads with >50% of low-quality bases in a single read.

### Mapping reads to human genome

Reference sequences were downloaded from the UCSC website (version hg19, http://genome.ucsc.edu/). Clean reads were respectively aligned to reference genomes and transcriptomes using Hisat2^20^. Output SAM files were converted to BAM files and sorted according to index. Duplicate reads were removed using Picard (http://broadinstitute.github.io/picard/). Only unique paired reads were used in final analysis.

### Differentially expressed gene analysis

HTSeq^21^ was used to generate the count matrix with the following parameters: ‘htseq-count -s no -i gene_name’ with the same GTF file used for the alignment step. The reference GTF file was downloaded from the GenCode website (version v27lift37, http://www.gencodegenes.org/). Default parameters of DEseq2^22^ were used for differential gene expression analysis.

FPKM method was used to calculating the expression level. The FPKM method was able to eliminate the influence of different gene length and sequencing discrepancy on the calculation of gene expression level. Therefore, the FPKM values can be directly used for comparing the differences of gene expression among groups. A fold change ≥2 and false discovery rate ≤0.05 were regarded as thresholds to identify DEGs.

### Detecting human gene fusions

The STAR-Fusion was used to detect gene fusions based on the paired-end reads of different samples

The STAR-Fusion parameters included: ‘STAR-Fusion-Fusion Inspector inspect–examine_coding_effect–annotate’. The results annotated as INFRAME and FRAMESHIFT were carried forward for further analysis^23^.

### PCR validation of fusion genes

Total RNA was extracted from the 9 samples. First-strand cDNA was synthesized according to total RNA by using FastQuant RT Kit (Tiangen). Reverse transcription was performed at 65 °C for 5 min and followed at 42 °C for 15 min to inactivate the reaction.

Primers of 11 in-frame or frameshift fusion genes which were used in PCR reaction were shown in Supplemental Table S4. Real-time PCR was performed with PCR Mix (Frpon). Real-time PCR was performed on an initial incubation at 50 °C for 2 min and then at 95 °C for 10 min, followed by 95 °C for 15 s with 40 repeat cycles, and finally at 60 °C for 30 s.

### Data access

The raw sequencing data for the 9 gliomas were deposited in the NCBI Sequence Read Archive (SRA) database and the corresponding accession numbers are (SRR6674299; SRR6674301; SRR6674302; SRR6674304; SRR6674303; SRR6674297; SRR6674298; SRR6674305; SRR6674300).

## Supplementary information

Identification of differentially expressed genes and fusion genes associated with malignant progression of spinal cord gliomas by transcriptome analysis

## Data Availability

All data and constructs are available upon request.

## References

[1] Hsu S (2011). Incidence patterns for primary malignant spinal cord gliomas: a Surveillance, Epidemiology, and End Results study. J. Neurosurg..

[2] Fakhreddine MH (2013). Treatment, prognostic factors, and outcomes in spinal cord astrocytomas. Neuro Oncol.

[3] Seki T (2015). Surgical outcomes of high-grade spinal cord gliomas. Asian Spine J..

[4] Louis DN, Ohgaki H, Wiestler OD, Cavenee WK (2007). The 2007 WHO classification of tumours of the central nervous system. Acta Neuropathol.

[5] Louis DN (2016). The 2016 World Health Organization classification of tumors of the central nervous system: a summary. Acta Neuropathol..

[6] Ostrom QT (2015). CBTRUS Statistical Report: Primary Brain and Central Nervous System Tumors Diagnosed in the United States in 2008-2012. Neuro-oncology.

[7] Jiang T (2016). CGCG clinical practice guidelines for the management of adult diffuse gliomas. Cancer letters.

[8] Cabili MN (2011). Integrative annotation of human large intergenic noncoding RNAs reveals global properties and specific subclasses. Gene dev..

[9] Ozsolak F, Milos PM (2011). RNA sequencing: advances, challenges and opportunities. Nat Rev Genet.

[10] Li Y (2013). Distinct genomic aberrations between low-grade and high-grade gliomas of Chinese patients. Plos One.

[11] Wei BO (2015). Identification of differentially expressed genes regulated by transcription factors in glioblastomas by bioinformatics analysis. Mol Med Rep..

[12] Xu Y (2017). Screening critical genes associated with malignant glioma using bioinformatics analysis. Mol Med Rep.

[13] Berger A (2003). Galanin and galanin receptors in human gliomas. Acta Neuropathol.

[14] Mannaioni G, Marino MJ, Valenti O, Traynelis SF, Conn PJ (2001). Metabotropic glutamate receptors 1 and 5 differentially regulate CA1 pyramidal cell function. J Neuropath..

[15] Yuan Yang, Jiaoming Li, Xiang Wang, Yanhui Liu, Shu Jiang, Maling Gou, Qing Mao (2018). Analyzing the interactions of mRNAs, miRNAs, lncRNAs and circRNAs to predict competing endogenous RNA networks in glioblastoma. Journal of Neuro-Oncology.

[16] Bao ZS (2014). RNA-seq of 272 gliomas revealed a novel, recurrent PTPRZ1-MET fusion transcript in secondary glioblastomas. Genome Res.

[17] Jones DTW (2009). Oncogenic RAF1 rearrangement and a novel BRAF mutation as alternatives to KIAA1549: BRAF fusion in activating the MAPK pathway in pilocytic astrocytoma. Oncogene..

[18] Lin A (2012). BRAF alterations in primary glial and glioneuronal neoplasms of the central nervous system with identification of 2 novel KIAA1549: BRAF fusion variants. J Neuropath Exp Neur..

[19] Kaul A, Chen YH, Emnett RJ, Dahiya S, Gutmann DH (2012). Pediatric glioma-associated KIAA1549: BRAF expression regulates neuroglial cell growth in a cell type-specific and mTOR-dependent manner. Gene dev..

[20] Kim D, Langmead B, Salzberg SL (2015). HISAT: a fast spliced aligner with low memory requirements. Nat Methods..

[21] Anders S, Pyl PT, Huber W (2015). HTSeq-a Python framework to work with high-throughput sequencing data. Bioinformatics.

[22] Love MI, Huber W, Anders S (2014). Moderated estimation of fold change and dispersion for RNA-seq data with DESeq2. Genome Biol..

[23] McKenna A (2010). The Genome Analysis Toolkit: a MapReduce framework for analyzing next-generation DNA sequencing data. Genome Res..

