# Supplementary material for: Identification of differentially expressed genes and fusion genes associated with malignant progression of spinal cord gliomas by transcriptome analysis
Source: Sci Rep. 2019 Sep 19;9:13583. doi: 10.1038/s41598-019-50072-9 (PMC6753211; doi:10.1038/s41598-019-50072-9)
Supplement: Supplementary file 1 — Identification of differentially expressed genes and fusion genes associated with malignant progression of spinal cord gliomas by transcriptome analysis [file 41598_2019_50072_MOESM1_ESM.pdf]

Supplementary information

Identification of differently expressed genes, fusion genes and mutated genes associated with the malignant progression of spinal cord gliomas by transcriptome analysis

Authors:
Dong-kang Liu[1], Jin Wang[1], Yi Guo[1], Zheng-xing Sun[1], Gui-huai Wang[1,2]

Addresses:
[1]Department of neurosurgery, Beijing Tsinghua Changgung Hospital, 102218, China;
[2]School of Clinical Medicine, Tsinghua University, Beijing, 100084, China.

Corresponding author: Gui-huai Wang
E-mail address: neurospinewgh@163.com)

Supplementary Table S1 Characteristics of libraries mapped to reference gene database

Sample ID	Raw reads	Clean reads	Total mapped	Multiple mapped	Uniquely mapped
S01 (grade Ⅱ)	26374768	18281210	16581447 (90.7%)	671553 (3.67%)	15909894 (87.03%)
S02 (grade Ⅱ)	29287160	18308306	16430308 (89.74%)	627639 (3.43%)	15802669 (86.31%)
S03 (grade Ⅱ)	34812436	23540518	21447731 (91.11%)	851822 (3.62%)	20595909 (87.49%)
S04 (grade Ⅱ)	26773424	14352260	12497000 (87.07%)	559883 (3.9%)	11937117 (83.17%)
S05 (grade Ⅳ)	33204894	18916628	16046711 (84.83%)	646156 (3.42%)	15400555 (81.41%)
S06 (grade Ⅳ)	31100538	20312198	18626061 (91.7%)	779340 (3.84%)	17846721 (87.86%)
S07 (grade Ⅳ)	26275874	14021924	11365194 (81.05%)	474856 (3.39%)	10890338 (77.67%)
S08 (grade Ⅳ)	32218992	20453534	18004262 (88.03%)	940983 (4.6%)	17063279 (83.42%)
S09 (grade Ⅳ)	24513220	14860896	12943461 (87.1%)	505235 (3.4%)	12438226 (83.7%)

Note: Sample ID: Sample name;

Raw reads: The number of reads in the raw data;

Clean reads: The number of reads filtered from the raw data;

Total mapped: The number of reads mapped to the genome;

Multiple mapped: Reads number compared to multiple locations of the genome;

Uniquely mapped: Number of reads mapped to a single location of the genome;

Supplementary Table S2 Sum of differentially expressed genes in Ⅳ vs Ⅱ.

Gene	Log_2 FoldChange Ⅳ vs Ⅱ	*P* value	FDR-adjusted *P*-Value
DSC3	-7.644464873	9.2747E-21	2.55E-16
INSM1	-4.757408157	5.60485E-16	7.72E-12
HCG11	4.048435957	2.85798E-15	2.62E-11
COL21A1	5.848582591	2.61561E-14	1.80E-10
ANGPTL1	-3.396304942	8.39226E-14	4.62E-10
TTC12	3.339669539	1.55741E-12	7.15E-09
MIR663AHG	-8.680383152	2.42055E-12	9.44E-09
TP53TG3D	-5.185378151	2.74279E-12	9.44E-09
VPS37B	2.81244086	5.21042E-12	1.49E-08
AC003973.3	-6.749704046	5.39692E-12	1.49E-08
PDIA2	-2.789790546	6.46502E-12	1.62E-08
TEX14	-3.49220684	1.02705E-11	2.36E-08
NTS	-7.364034876	5.96609E-11	1.26E-07
LINC01098	-7.384175145	7.5591E-11	1.48E-07
PLAG1	-3.33033095	8.06609E-11	1.48E-07
AC107952.2	-3.020672634	1.42343E-10	2.45E-07
PDLIM4	4.04282121	4.28315E-10	6.94E-07
ZNF560	-4.6081876	4.65791E-10	7.13E-07
AL121904.1	-8.503642103	2.58352E-09	3.74E-06
MTHFD2	-2.184382432	3.13129E-09	4.31E-06
CERS3-AS1	-7.124660409	3.92797E-09	5.05E-06
SALL2	2.042423998	4.03736E-09	5.05E-06
NPPA	-5.117147686	5.13175E-09	6.14E-06
CD163L1	-3.916580658	5.58061E-09	6.40E-06
PAX3	-3.091501649	8.24707E-09	9.08E-06
PLEKHG4	-2.993476157	8.78503E-09	9.31E-06
ELOVL2	2.807287313	9.6495E-09	9.82E-06
AC012593.1	6.116733733	9.9855E-09	9.82E-06
TCEA3	2.50141056	1.58874E-08	1.51E-05
AC002351.1	-5.367016888	1.71192E-08	1.57E-05
AC022893.2	-4.572351569	2.69809E-08	2.40E-05
PRAME	-9.147589372	2.96841E-08	2.55E-05
HERC2P4	-4.813024781	3.50193E-08	2.92E-05
OR5AK4P	3.979787313	3.76244E-08	3.05E-05
TMEM229A	3.331206486	4.89054E-08	3.85E-05
IQGAP2	-2.867257858	7.00629E-08	5.36E-05
AL359233.1	3.906260429	8.56009E-08	6.37E-05
ZFHX4	4.231190322	1.10891E-07	8.04E-05

AP002840.2	5.046766807	1.27343E-07	8.99E-05
AC016717.2	-4.694015645	1.60249E-07	0.000110327
ZNF208	-5.133979464	1.64882E-07	0.000110748
BEAN1	-2.862682419	1.69141E-07	0.000110904
CPXM2	3.831338999	2.32447E-07	0.000148869
GPR17	5.148566557	2.46582E-07	0.000154332
CCDC8	3.441994921	2.67014E-07	0.000163407
DMKN	4.802693541	2.73979E-07	0.000164024
PAPPA2	-3.223563981	2.83056E-07	0.000164735
MAB21L1	-3.838732851	2.87131E-07	0.000164735
AC008556.1	2.416191027	3.74578E-07	0.000210521
PNPLA4	2.853256819	4.08164E-07	0.000224808
NPY5R	-3.656681678	4.31307E-07	0.000228688
LINC01993	-5.14714583	4.31817E-07	0.000228688
ARAP3	-2.749019938	4.70472E-07	0.000244459
ALX3	-7.154724083	5.2235E-07	0.000266389
LIMS2	4.163685291	5.37501E-07	0.000267441
ZFHX4-AS1	5.083927683	5.43835E-07	0.000267441
GRM5	4.10187018	6.13001E-07	0.000296166
TKTL1	-4.500305761	6.84177E-07	0.000324854
FABP5	2.736760314	7.02857E-07	0.000328068
AL512380.1	-4.944123346	7.15801E-07	0.000328541
LINC01436	-5.053067136	7.50135E-07	0.000338655
CNN2P8	5.322943855	8.16575E-07	0.000362704
FILIP1L	-2.352645647	9.88889E-07	0.000429082
LINC00944	-6.012477291	9.97178E-07	0.000429082
AC104041.1	-6.105182941	1.10744E-06	0.000468092
TP63	-4.569705164	1.12183E-06	0.000468092
MRO	2.69626528	1.16117E-06	0.000477276
FRMD6	1.937745695	1.26201E-06	0.000511096
ANKRD34C	5.702175037	1.3833E-06	0.000552096
CACNB3	-2.450926087	1.45727E-06	0.000573311
SPX	3.766180047	1.61298E-06	0.000625632
CNMD	3.730431934	1.64038E-06	0.000627424
AL355974.3	-3.751578875	1.75819E-06	0.000663271
RAB34	2.785875496	1.87693E-06	0.000698497
GLIS1	-2.647677692	1.95661E-06	0.00071844
WSCD2	3.721535607	1.99745E-06	0.000723785
FAM162B	-2.636745292	2.35023E-06	0.00084056
OSR1	-4.768696952	2.44743E-06	0.000857315
ACSM4	-4.735939035	2.45934E-06	0.000857315
NPY2R	-6.927528924	2.50691E-06	0.000862973
HSPA2	3.115854541	2.57299E-06	0.000869136
MUC12	-3.775541276	2.59765E-06	0.000869136

LIF	-3.056087175	2.6195E-06	0.000869136
AC109466.1	-6.734968879	2.90094E-06	0.000951059
NMU	-4.878662637	3.05069E-06	0.000987559
PCDHGA9	4.072507508	3.08399E-06	0.000987559
TBX5	5.891410841	3.12977E-06	0.000990697
GPD1	2.749639667	3.33804E-06	0.001044618
MGAT5B	-1.98115571	3.47755E-06	0.001076048
AC138761.4	-7.145596302	3.73913E-06	0.001144132
A2MP1	1.493783104	4.31055E-06	0.001304487
AC100863.1	-5.630074048	4.409E-06	0.001319777
CCT7P2	3.622224281	4.58626E-06	0.001341594
RP11-435B5.5	-4.731831825	4.62139E-06	0.001341594
GALR1	-2.986850688	4.62803E-06	0.001341594
COL5A1	-3.236190007	4.88889E-06	0.001402449
COPZ2	2.45764328	4.95963E-06	0.001408075
PRICKLE3	2.281443846	5.08847E-06	0.001429912
MFAP2	-3.081022594	5.40668E-06	0.001503987
RPS28P7	-2.377599865	5.49237E-06	0.001512545
APC	1.286704968	5.61789E-06	0.001531794
CHSY1	-1.543770817	6.14144E-06	0.001658129
AC010636.1	-6.53741555	6.29978E-06	0.001669941
LINC00870	4.640031031	6.30647E-06	0.001669941
AL845331.1	4.645493422	6.45725E-06	0.001693583
AC021218.1	-5.402962907	6.54571E-06	0.001700588
AC073365.1	-9.088799312	6.75998E-06	0.001739842
CHDH	1.477829771	7.0082E-06	0.001787026
BSPRY	3.314865659	7.29544E-06	0.001829074
DLG5-AS1	-1.919400462	7.33662E-06	0.001829074
HPSE2	4.969459944	7.37235E-06	0.001829074
CLEC2B	-2.176180598	7.7678E-06	0.001909978
RHOD	3.211622741	8.0926E-06	0.001972232
NRN1	3.622332004	8.8322E-06	0.002133596
FABP5P7	2.77419541	8.94595E-06	0.002142282
SLC39A12	3.351338244	9.06446E-06	0.00215195
NPAS1	4.122628687	9.20431E-06	0.002166474
SPOCD1	-3.956560042	9.82323E-06	0.00229256
NPY1R	-2.117313312	1.00139E-05	0.002317425
SLC43A2	1.814651239	1.04447E-05	0.00239697
DNAJC15	1.269704813	1.12576E-05	0.002562168
AGTR1	-5.170728093	1.18406E-05	0.00267278
SPTSSB	-3.195173809	1.23227E-05	0.002758979
FKBP5	-3.909012656	1.25845E-05	0.002794867
SIGLEC1	-2.882725491	1.28938E-05	0.002825899
ACOT1	2.431159478	1.29294E-05	0.002825899

AC073578.2	-4.879862715	1.34528E-05	0.00291713
MTND1P23	-6.437867426	1.41355E-05	0.003041232
LINC02094	-3.408775539	1.57807E-05	0.003345308
LINC01257	-5.33304191	1.58814E-05	0.003345308
CBX2	-2.68419043	1.59133E-05	0.003345308
CXCL10	-3.387200186	1.65208E-05	0.003446718
BEX2	1.801355602	1.67899E-05	0.00347652
BCL11A	-3.294846315	1.70709E-05	0.003508334
LINC00900	1.966832175	1.73059E-05	0.003530283
HSF4	-2.158418587	1.76127E-05	0.003566445
SAMD14	-1.64610371	1.79426E-05	0.003606729
STXBP6	1.879392512	1.82002E-05	0.003622027
LGSN	-4.513488141	1.82818E-05	0.003622027
SERPINE1	-2.844319491	1.96211E-05	0.003859601
GSX1	-3.653817453	2.02564E-05	0.00395632
AC108156.1	5.650797199	2.24583E-05	0.004331141
OCIAD2	-2.464085196	2.26165E-05	0.004331141
SVEP1	-3.235852787	2.27874E-05	0.004331141
MUM1L1	-2.241059001	2.28046E-05	0.004331141
TMEM184A	-4.493275624	2.45048E-05	0.004622177
TMSB10	-1.433457103	2.54658E-05	0.004770774
DNM1	-1.557955363	2.58555E-05	0.00481104
AC112255.1	-6.432565448	2.67073E-05	0.004919971
AC027290.2	2.36600108	2.69298E-05	0.004919971
LINC02388	-5.556839856	2.69769E-05	0.004919971
DRAXIN	-2.938571849	2.74469E-05	0.004972762
C6orf141	-4.477223764	2.80134E-05	0.005022783
AF279873.3	-4.658063865	2.80878E-05	0.005022783
MROH3P	3.256099892	3.05796E-05	0.00543311
FABP6	-3.68180446	3.08902E-05	0.005453114
PDLIM1	-2.945507139	3.17195E-05	0.005504303
BMP7	1.906375184	3.17755E-05	0.005504303
GYPE	-2.34190573	3.17798E-05	0.005504303
LINC00639	2.395847562	3.28555E-05	0.005655056
C2CD4C	-2.383600399	3.30988E-05	0.005661536
CLVS2	4.118044393	3.48813E-05	0.0059296
MMP23B	-3.695747026	3.54976E-05	0.005997354
ERVW-1	1.731842783	3.6021E-05	0.006037212
HOXB5	1.878242281	3.6172E-05	0.006037212
EMX2OS	2.98752369	3.69428E-05	0.006128722
EMX2	3.146923035	3.74421E-05	0.006173335
DLK1	-7.924022069	3.76601E-05	0.006173335
B4GALNT1	-2.30361355	4.38385E-05	0.007143594
GPD1L	2.450590631	4.47442E-05	0.00721667

DNAH3	-3.665192461	4.48163E-05	0.00721667
NR3C2	2.595548419	4.50731E-05	0.00721667
AP000525.10	-3.412986557	4.68682E-05	0.00746071
GPR37L1	2.868855656	4.73541E-05	0.007494734
AC022215.1	-3.042312805	4.77844E-05	0.007519628
RASD1	-3.351039617	4.90338E-05	0.007672398
AC006946.3	-2.732593984	5.14534E-05	0.008005504
CRYGD	-3.756098054	5.47911E-05	0.008440118
SYT7	-2.735527023	5.48597E-05	0.008440118
WNT6	-4.2294029	5.57359E-05	0.008527289
AC006548.3	-3.444037561	5.61748E-05	0.008546943
AC099654.7	-5.942090208	5.8194E-05	0.00880552
KCNS1	3.539331534	5.92269E-05	0.00891284
LINC00346	-2.865412172	6.05361E-05	0.009060339
SCN5A	-3.389938577	6.33631E-05	0.009432203
TNFRSF12A	-1.816032417	6.40089E-05	0.009435857
PCDHGC4	4.081426323	6.44991E-05	0.009435857
NPSR1-AS1	-5.347191345	6.47053E-05	0.009435857
SLC6A15	-3.634437722	6.47971E-05	0.009435857
GSX2	-3.57899244	6.51009E-05	0.009435857
KHDRBS2	-3.552391119	6.71771E-05	0.009685815
PCDHGC3	3.450061259	6.95072E-05	0.009947335
TLR10	2.740048268	6.97133E-05	0.009947335
ACOT2	1.348741288	7.09435E-05	0.010057304
AC005220.1	4.08690331	7.12144E-05	0.010057304
SLITRK5	1.326492477	7.22384E-05	0.010149861
HOXB7	1.983641441	7.45504E-05	0.010421535
TNFRSF18	-2.486586367	7.60124E-05	0.010572251
STK26	-2.368553784	7.87033E-05	0.010845459
JADE1	-1.334598177	7.87644E-05	0.010845459
PCA3	-2.983461013	7.97887E-05	0.010931844
MYO7A	-2.312450221	8.28123E-05	0.011289936
LINC01170	3.447144926	8.39522E-05	0.011388958
AC022905.1	-4.795460462	8.50185E-05	0.011477075
HLA-F	-4.880337682	8.68899E-05	0.011639023
BCAS1	3.113002467	8.75285E-05	0.011639023
HOXB-AS2	2.967564223	8.75816E-05	0.011639023
MOV10L1	2.136691132	8.79355E-05	0.011639023
AC040160.1	-2.269562401	8.83313E-05	0.011639023
AP001372.1	-2.858658204	9.12E-05	0.011959799
KRT18P14	-3.160451216	9.16663E-05	0.011963968
SNORD3B-2	-3.74735665	9.90954E-05	0.012872588
KCNB2	-4.785326382	9.98768E-05	0.012913179
RNU2-57P	-6.045551001	0.000102031	0.013130109

AL139393.2	-2.737111897	0.000104213	0.013243384
SOWAHA	2.399463172	0.000104258	0.013243384
PSMC1P7	-4.742793634	0.000104354	0.013243384
GADD45A	-2.377299928	0.000105753	0.013359327
ITPR2	1.57304713	0.000107934	0.013552805
ACBD7	2.99762363	0.00010853	0.013552805
AL109924.4	-4.473908192	0.000108761	0.013552805
MGAM	-2.328648574	0.00011112	0.013784375
HSD17B6	2.730204159	0.000112973	0.013951423
GAP43	-2.26486927	0.000115538	0.014204439
HS3ST3A1	-4.460078887	0.000116277	0.014231768
AC007681.1	-1.997136562	0.000118242	0.014368883
AL109924.2	-4.47356612	0.000118539	0.014368883
PRODH	2.168578578	0.000118962	0.014368883
RGS20	2.745971632	0.000119506	0.014371495
UBL4B	-5.010769611	0.000120379	0.014413532
XK	1.54164891	0.000123675	0.014744059
IL36G	-5.172160671	0.00012622	0.014982664
NOS2	-1.663714216	0.000130438	0.01536706
AQP7P2	-4.359000364	0.000130724	0.01536706
AP003900.1	-9.724557949	0.000131133	0.01536706
FZD10-AS1	-2.825637462	0.000135628	0.015758105
EBF1	-2.267007974	0.000135914	0.015758105
ENTPD2	3.515811129	0.000136186	0.015758105
GNRH1	-1.887017916	0.000138916	0.016006712
Z73979.1	-3.042659485	0.000141743	0.016264452
ATP2B3	3.412077788	0.000142409	0.016273075
EXTL1	1.929599317	0.000146868	0.016713257
TENM2	-2.934680186	0.000148112	0.016713661
CIPC	1.512977644	0.000148452	0.016713661
PGR	2.745098119	0.000148693	0.016713661
AC015712.6	-2.30657579	0.000153082	0.017137133
LL22NC03-N64E9.1	-4.332723457	0.000156145	0.017409214
AC092652.1	5.729327765	0.000157139	0.017449435
PCDHGC5	2.496088166	0.000158609	0.017500532
ERI2	1.176714944	0.00015887	0.017500532
FAM160A1	-5.136766007	0.000160546	0.017614682
FREM3	-3.65706256	0.000166361	0.018180251
BIRC7	-2.867909262	0.000167728	0.018257109
CNGB1	-6.149890342	0.000169499	0.01837725
TSPAN2	-2.752825626	0.000175839	0.018982422
AL031728.1	-2.433397618	0.000176459	0.018982422
BANCR	-4.094502173	0.000179876	0.019274743
AC107375.1	-1.197009306	0.000181675	0.019392006

PCDHA11	2.848181708	0.000183629	0.019524975
MAGEC2	-7.968210488	0.000186563	0.019760557
AC145098.2	-2.208041003	0.000187298	0.019762484
NYAP2	-4.082996618	0.000192352	0.020218259
GABRE	-2.487780459	0.000194767	0.020394237
CLIC5	1.995243061	0.000198968	0.020755187
CASZ1	-2.441336768	0.000204451	0.021246701
NPC1L1	-2.961674862	0.000206315	0.021359849
AC008554.1	-3.983514984	0.000208035	0.021457215
LINC00871	-5.358204506	0.000209965	0.021575503
BEAN1-AS1	-3.228103654	0.000214311	0.021896648
HIRAP1	-3.605382397	0.000214681	0.021896648
MEGF10	2.151133403	0.000217707	0.022123383
AC008708.1	-5.453672264	0.000223953	0.02267441
LDHB	1.527915183	0.000234176	0.023548782
CAB39L	1.55738958	0.000234588	0.023548782
FGF13	-1.594590977	0.00023576	0.023548782
PIN4P1	-2.886581191	0.000236009	0.023548782
EPOR	1.359105489	0.000239111	0.023772106
BX255923.3	-3.463643935	0.000245417	0.024276411
AC060766.1	2.806481709	0.000245946	0.024276411
AC004877.1	2.92905524	0.000247951	0.024386874
PPP1R16B	2.428561335	0.000251281	0.024531604
STX8P1	-2.500614108	0.000251516	0.024531604
RINL	1.865257067	0.00025215	0.024531604
ATP1A2	2.589111897	0.000252986	0.024531604
RDH10	-1.53154111	0.000257265	0.024772075
OR7E115P	5.201241917	0.000258952	0.024822834
AC017006.2	-3.085818938	0.000260349	0.024822834
LINC01060	-5.764420013	0.000260496	0.024822834
AC136424.2	-3.380664538	0.000267817	0.025345406
GAL	-4.192149813	0.000268375	0.025345406
FOXF2	1.794376037	0.000268741	0.025345406
AC011195.2	-5.059456383	0.000270307	0.025406088
LL22NC03-N14H11.1	-3.454807382	0.000277948	0.026035365
HAGLROS	2.748682983	0.000278932	0.02603902
AP003119.3	-2.269643835	0.00028193	0.026201945
IGHV3-9	6.237014823	0.000283135	0.026201945
FGF13-AS1	-2.67206446	0.000284347	0.026201945
TDRD9	3.298061077	0.000285267	0.026201945
DUSP5P1	-3.065241725	0.000285656	0.026201945
CITED1	-3.014429331	0.000286386	0.026201945
ERMP1	1.278817831	0.000289406	0.026390593
MICA	2.682244157	0.00029153	0.026496495

AC092112.1	-3.142721798	0.000292814	0.026525629
MT1G	3.665337323	0.000294722	0.026610998
GJB6	6.194612287	0.000297907	0.026810665
C17orf67	-1.713445595	0.000320513	0.028751184
MTSS1L	1.625864702	0.000321753	0.028768714
LIN7A	-1.622614285	0.000326415	0.029091113
EFNA5	-3.747240945	0.000333239	0.029603462
PWRN1	-3.463571147	0.00033572	0.029643301
AC022113.1	-2.151622225	0.000336035	0.029643301
CHCHD2P11	-4.446998958	0.00033753	0.029643301
GLT1D1	-1.824932363	0.000337993	0.029643301
DSG2	-3.386520571	0.000342497	0.029942938
AC016044.1	5.988822729	0.000343856	0.029966637
PNCK	2.182920915	0.000345293	0.029996885
AC068987.3	-3.750669577	0.000347105	0.030059501
CCNYL2	-3.066793003	0.000350915	0.030248219
AC018695.7	4.848871869	0.000351481	0.030248219
HMGA1P7	6.242618376	0.000354118	0.030380277
LINC01405	-3.925305003	0.000356004	0.030447154
SCARF2	-1.661394647	0.000359741	0.030671548
ELAVL4	-2.325372411	0.000367775	0.031259716
AC093642.2	2.923813657	0.000388673	0.032934319
KRT16P2	-7.571760694	0.000390064	0.03295084
RSPO3	-3.597557925	0.000392118	0.033023011
SNORD124	-5.084888543	0.000400462	0.033538941
LINC02058	3.452324622	0.000401699	0.033538941
AC078917.1	-5.115471492	0.000402616	0.033538941
GPR161	-1.090718872	0.000403587	0.033538941
LINC02100	-2.667363744	0.000404333	0.033538941
ACAA2	1.298204011	0.000411733	0.034050199
ANKRD28	1.156119363	0.000422876	0.034867047
CCNA1	-3.058787455	0.000425885	0.035010305
FOXQ1	2.327984883	0.000427774	0.035041206
ALDH6A1	1.464744058	0.000428806	0.035041206
COL24A1	-3.338398779	0.000431795	0.035171743
AC093895.1	-4.176665931	0.000432958	0.035171743
UPK3A	3.028871934	0.000436218	0.035309435
AACSP1	-4.471332115	0.000437677	0.035309435
IGFBP3	-3.155122921	0.000438499	0.035309435
SEPT7P3	2.902712767	0.000440168	0.03534048
NNMT	-3.561347309	0.000443672	0.035401024
CHADL	2.167714384	0.000444408	0.035401024
KLF6	-1.094197598	0.000444778	0.035401024
PCDHA4	3.134398727	0.000448959	0.035630808

PLPP4	1.163189491	0.000451911	0.035706526
IGF2BP2-AS1	-2.403379259	0.000452507	0.035706526
FBN2	-2.517646438	0.000459283	0.036137735
PDE10A	-2.043510665	0.000471367	0.036877771
FAM84B	-1.39580359	0.000473434	0.036934554
AC080100.1	1.49847605	0.000475087	0.036958799
LINC01829	-4.651596088	0.000482068	0.037396291
FBXL22	1.534961982	0.000490516	0.037944702
MIR5690	-4.792159491	0.000493016	0.038031305
LINC01140	2.530813954	0.000500009	0.038462999
HHIP-AS1	-1.729221196	0.000515983	0.039581205
GJB2	3.026247291	0.000521139	0.039831892
TEX22	1.620395508	0.00052268	0.039831892
LINC01561	1.387266819	0.00052426	0.039831892
IP6K3	-2.741733677	0.000525036	0.039831892
EDARADD	-2.571972833	0.000526853	0.039859918
AP001521.1	4.933134532	0.000538828	0.040654197
HLA-A	-5.581744431	0.000541247	0.040725151
CEL	-1.790452862	0.000549527	0.041235471
ISYNA1	-1.237400937	0.000555983	0.041520946
C8orf4	-2.303681602	0.000556347	0.041520946
RNF141	1.074970691	0.000558294	0.041553704
AC024451.4	-2.050352898	0.000564002	0.041816883
SLC47A1P1	-5.025964089	0.000565908	0.041816883
FGR	-1.448398265	0.000566386	0.041816883
PAH	2.778270873	0.000575488	0.042341617
LINC01679	-3.686348503	0.000576568	0.042341617
HIST1H2BH	-2.609847102	0.000584614	0.0427867
FAM50B	2.327432222	0.000586965	0.0427867
SORCS2	1.74592075	0.00058729	0.0427867
LINC01535	1.33461199	0.000597126	0.043388513
SLC25A5	1.517676479	0.000604647	0.043819385
FGF5	-4.734752983	0.000607309	0.043830486
AC004448.4	-2.138803399	0.000608119	0.043830486
METTL24	3.260404154	0.000609575	0.043830486
HOXB6	2.811615802	0.000616736	0.044158994
HACD3	1.476853425	0.00061735	0.044158994
AC104461.1	3.309378208	0.000627284	0.044753305
GPRC5A	-3.415520467	0.000633136	0.044962436
RP11-782C8.2	-3.337924452	0.000633481	0.044962436
CDH18	-2.305964548	0.000643266	0.045539587
LINC01750	-2.107347203	0.000651334	0.045992559
AL162731.1	-4.376203643	0.000654183	0.04607554
MDGA2	1.806972757	0.000657477	0.046114886

ALX1	-7.801851928	0.00065809	0.046114886
AC090692.1	-2.48181863	0.00065982	0.046118772
FCRLA	3.240039808	0.000663481	0.046257226
SGCD	1.502176983	0.000674355	0.046820027
AC007240.1	-3.50070573	0.000674954	0.046820027
AL354919.2	-5.278010553	0.000677284	0.046863645
CDK18	2.646107571	0.000686913	0.047410777
AC118942.1	-5.485185233	0.000695344	0.047872706
ASIC2	2.703498696	0.000704608	0.048271825
AC021683.3	-3.714653592	0.000704647	0.048271825
AC023794.3	2.442013728	0.000706434	0.04827417
AC006058.4	3.0081952	0.000711838	0.048523067
RPS3AP14	-2.65973728	0.000716734	0.048533634
AL160286.2	-3.179367667	0.000719349	0.048533634
AC009902.2	3.366317782	0.000720448	0.048533634
GPR135	1.152219671	0.000720805	0.048533634
APOL4	-2.23186537	0.000728605	0.048939173
AC020913.3	-2.354072807	0.000736052	0.049319041
TYRP1	-4.042966946	0.0007395	0.049377417
AC068491.4	-2.718734003	0.000742103	0.049377417
NAPEPLD	1.410854247	0.000743852	0.049377417
FA2H	3.018440872	0.000746911	0.049377417
KY	2.096585794	0.000747149	0.049377417
CADPS	-1.680534177	0.000747681	0.049377417
SEC11C	1.471961517	0.00075615	0.049762054
PITX1	-6.557106128	0.000757119	0.049762054

Note:

Log_2 FoldChange (Ⅳ vs Ⅱ): The multiple value of difference between two samples or comparison combinations, expressed as log_2 (group1/group2), calculated according to the difference analysis software

P value: *P* value of significance test

FDR-adjusted *P*-Value: *P* value of multiple hypothesis test correction

Supplementary Table S3 Pathway analysis of differentially expressed genes in Ⅳ vs Ⅱ.

Pathway ID	Description	*P* value	FDR-adjusted *P*-Value	Gene ID	Count
hsa04080	Neuroactive ligand-receptor interaction	0.007960251	0.39403243	NTS/NPY5R/GRM5/NPY2R/NMU/GALR1/ NPY1R/AGTR1/GNRH1/GABRE/GAL	11
hsa04218	Cellular senescence	0.00269618	0.177947873	SERPINE1/HLA-F/GADD45A/ITPR2/CCNA1/ IGFBP3/HLA-A/SLC25A5	8
hsa04022	cGMP-PKG signaling pathway	0.011902866	0.44819415	NPPA/AGTR1/ITPR2/ATP2B3/CNGB1/ ATP1A2/SLC25A5	7
hsa04024	cAMP signaling pathway	0.038984128	0.689750173	NPPA/ARAP3/NPY1R/ATP2B3/CNGB1/ ATP1A2/PDE10A	7
hsa04060	Cytokine-cytokine receptor interaction	0.09985891	0.689750173	LIF/CXCL10/BMP7/TNFRSF12A/TNFRSF18/ IL36G/EPOR	7
hsa04020	Calcium signaling pathway	0.064603668	0.689750173	GRM5/AGTR1/ITPR2/NOS2/ATP2B3/SLC25A5	6
hsa04144	Endocytosis	0.176337224	0.799081256	VPS37B/ARAP3/HSPA2/DNM1/HLA-F/ HLA-A	6
hsa00062	Fatty acid elongation	5.19E-05	0.010268165	ELOVL2/ACOT1/ACOT2/ACAA2/HACD3	5
hsa04925	Aldosterone synthesis and secretion	0.013581641	0.44819415	NPPA/AGTR1/ITPR2/ATP2B3/ATP1A2	5
hsa04261	Adrenergic signaling in cardiomyocytes	0.067382076	0.689750173	CACNB3/AGTR1/SCN5A/ATP2B3/ATP1A2	5
hsa05224	Breast cancer	0.072390199	0.689750173	APC/WNT6/GADD45A/PGR/FGF5	5
hsa04934	Cushing syndrome	0.083004586	0.689750173	APC/AGTR1/RASD1/WNT6/ITPR2	5
hsa04390	Hippo signaling pathway	0.090517673	0.689750173	FRMD6/APC/SERPINE1/BMP7/WNT6	5
hsa05167	Kaposi sarcoma-associated herpesvirus infection	0.128454983	0.748061373	CLEC2B/HLA-F/ITPR2/MICA/HLA-A	5
hsa05169	Epstein-Barr virus infection	0.157205345	0.799081256	CXCL10/HLA-F/GADD45A/CCNA1/HLA-A	5
hsa04010	MAPK signaling pathway	0.457200665	0.812762779	CACNB3/HSPA2/GADD45A/EFNA5/FGF5	5
hsa05165	Human	0.5466930	0.846330358	APC/WNT6/HLA-F/CCNA1/HLA-A	5

	papillomavirus infection	4			
hsa01040	Biosynthesis of unsaturated fatty acids	0.000747028	0.073955787	ELOVL2/ACOT1/ACOT2/HACD3	4
hsa04974	Protein digestion and absorption	0.040300319	0.689750173	COL21A1/COL5A1/ATP1A2/COL24A1	4
hsa04972	Pancreatic secretion	0.049929865	0.689750173	ITPR2/ATP2B3/ATP1A2/CEL	4
hsa04915	Estrogen signaling pathway	0.107208477	0.689750173	HSPA2/FKBP5/ITPR2/PGR	4
hsa04371	Apelin signaling pathway	0.15368691	0.799081256	AGTR1/SERPINE1/ITPR2/NOS2	4
hsa04723	Retrograde endocannabinoid signaling	0.168621549	0.799081256	GRM5/ITPR2/GABRE/NAPEPLD	4
hsa05226	Gastric cancer	0.187192803	0.799276255	APC/WNT6/GADD45A/FGF5	4
hsa04310	Wnt signaling pathway	0.222825788	0.812762779	PRICKLE3/APC/WNT6/RSPO3	4
hsa05202	Transcriptional misregulation in cancer	0.312632896	0.812762779	PAX3/GADD45A/CCNA1/IGFBP3	4
hsa05203	Viral carcinogenesis	0.351823623	0.812762779	HLA-F/CCNA1/HLA-A/HIST1H2BH	4
hsa05205	Proteoglycans in cancer	0.369686872	0.812762779	COL21A1/HPSE2/WNT6/ITPR2	4
hsa05166	Human T-cell leukemia virus 1 infection	0.412403555	0.812762779	HLA-F/CCNA1/HLA-A/SLC25A5	4
hsa05168	Herpes simplex virus 1 infection	0.9397386	0.944508847	ZNF560/ZNF208/HLA-F/HLA-A	4
hsa00830	Retinol metabolism	0.04056605	0.689750173	PNPLA4/HSD17B6/RDH10	3
hsa01212	Fatty acid metabolism	0.04934615	0.689750173	ELOVL2/ACAA2/HACD3	3
hsa05416	Viral myocarditis	0.04934615	0.689750173	HLA-F/HLA-A/SGCD	3
hsa04612	Antigen processing and presentation	0.064097253	0.689750173	HSPA2/HLA-F/HLA-A	3
hsa04924	Renin secretion	0.074934009	0.689750173	NPPA/AGTR1/ITPR2	3
hsa05217	Basal cell carcinoma	0.074934009	0.689750173	APC/WNT6/GADD45A	3

hsa04115	p53 signaling pathway	0.098827783	0.689750173	SERPINE1/GADD45A/IGFBP3	3
hsa05412	Arrhythmogenic right ventricular cardiomyopathy (ARVC)	0.102008832	0.689750173	CACNB3/DSG2/SGCD	3
hsa04970	Salivary secretion	0.118509363	0.71105618	ITPR2/ATP2B3/ATP1A2	3
hsa05222	Small cell lung cancer	0.17314178	0.799081256	GADD45A/NOS2/BIRC7	3
hsa00564	Glycerophospholipid metabolism	0.184828406	0.799276255	GPD1/GPD1L/PLPP4	3
hsa04066	HIF-1 signaling pathway	0.196719207	0.799276255	NPPA/SERPINE1/NOS2	3
hsa05145	Toxoplasmosis	0.225143758	0.812762779	HSPA2/NOS2/BIRC7	3
hsa04270	Vascular smooth muscle contraction	0.305396096	0.812762779	NPPA/AGTR1/ITPR2	3
hsa04514	Cell adhesion molecules (CAMs)	0.352627588	0.812762779	SIGLEC1/HLA-F/HLA-A	3
hsa04550	Signaling pathways regulating pluripotency of stem cells	0.361197546	0.812762779	LIF/APC/WNT6	3
hsa04072	Phospholipase D signaling pathway	0.38680085	0.812762779	GRM5/AGTR1/DNM1	3
hsa04921	Oxytocin signaling pathway	0.403745609	0.812762779	NPPA/CACNB3/ITPR2	3
hsa05225	Hepatocellular carcinoma	0.478060836	0.823096049	APC/WNT6/GADD45A	3
hsa05016	Huntington disease	0.505836664	0.831854934	GRM5/DNAH3/SLC25A5	3
hsa04360	Axon guidance	0.536641695	0.846330358	RHOD/BMP7/EFNA5	3
hsa05170	Human immunodeficiency virus 1 infection	0.591392246	0.869717016	HLA-F/ITPR2/HLA-A	3
hsa04015	Rap1 signaling pathway	0.601839913	0.869717016	ARAP3/EFNA5/FGF5	3
hsa04810	Regulation of actin cytoskeleton	0.628846048	0.881084419	IQGAP2/APC/FGF5	3
hsa05163	Human cytomegalovirus infection	0.635400709	0.881084419	HLA-F/ITPR2/HLA-A	3

hsa04151	PI3K-Akt signaling pathway	0.894976831	0.939502295	EPOR/EFNA5/FGF5	3
hsa00534	Glycosaminoglycan biosynthesis - heparan sulfate / heparin	0.05329203	0.689750173	HS3ST3A1/EXTL1	2
hsa05330	Allograft rejection	0.08374233	0.689750173	HLA-F/HLA-A	2
hsa05332	Graft-versus-host disease	0.08374233	0.689750173	HLA-F/HLA-A	2
hsa00640	Propanoate metabolism	0.088445033	0.689750173	LDHB/ALDH6A1	2
hsa04940	Type I diabetes mellitus	0.103001161	0.689750173	HLA-F/HLA-A	2
hsa04960	Aldosterone-regulated sodium reabsorption	0.107991189	0.689750173	NR3C2/ATP1A2	2
hsa04975	Fat digestion and absorption	0.107991189	0.689750173	NPC1L1/CEL	2
hsa05320	Autoimmune thyroid disease	0.118158683	0.71105618	HLA-F/HLA-A	2
hsa04973	Carbohydrate digestion and absorption	0.144529334	0.799081256	MGAM/ATP1A2	2
hsa04961	Endocrine and other factor-regulated calcium reabsorption	0.160891655	0.799081256	DNM1/ATP1A2	2
hsa00280	Valine, leucine and isoleucine degradation	0.171981467	0.799081256	ACAA2/ALDH6A1	2
hsa00330	Arginine and proline metabolism	0.177573613	0.799081256	PRODH/NOS2	2
hsa04978	Mineral absorption	0.177573613	0.799081256	ATP1A2/MT1G	2
hsa04923	Regulation of lipolysis in adipocytes	0.194511787	0.799276255	NPPA/NPY1R	2
hsa00561	Glycerolipid metabolism	0.22892813	0.812762779	PLPP4/CEL	2
hsa05213	Endometrial cancer	0.22892813	0.812762779	APC/GADD45A	2
hsa04927	Cortisol synthesis and secretion	0.240504025	0.812762779	AGTR1/ITPR2	2

hsa04720	Long-term potentiation	0.263729014	0.812762779	GRM5/ITPR2	2
hsa04918	Thyroid hormone synthesis	0.281163442	0.812762779	ITPR2/ATP1A2	2
hsa05218	Melanoma	0.286970322	0.812762779	GADD45A/FGF5	2
hsa01230	Biosynthesis of amino acids	0.310140338	0.812762779	TKTL1/PAH	2
hsa04971	Gastric acid secretion	0.310140338	0.812762779	ITPR2/ATP1A2	2
hsa04260	Cardiac muscle contraction	0.315912443	0.812762779	CACNB3/ATP1A2	2
hsa04742	Taste transduction	0.315912443	0.812762779	ENTPD2/ASIC2	2
hsa00562	Inositol phosphate metabolism	0.321674114	0.812762779	ALDH6A1/ISYNA1	2
hsa03320	PPAR signaling pathway	0.321674114	0.812762779	FABP5/FABP6	2
hsa05410	Hypertrophic cardiomyopathy (HCM)	0.378497669	0.812762779	CACNB3/SGCD	2
hsa04540	Gap junction	0.384081113	0.812762779	GRM5/ITPR2	2
hsa05210	Colorectal cancer	0.389643406	0.812762779	APC/GADD45A	2
hsa04912	GnRH signaling pathway	0.400701767	0.812762779	ITPR2/GNRH1	2
hsa05414	Dilated cardiomyopathy (DCM)	0.400701767	0.812762779	CACNB3/SGCD	2
hsa05032	Morphine addiction	0.406196515	0.812762779	GABRE/PDE10A	2
hsa04922	Glucagon signaling pathway	0.433301796	0.812762779	ITPR2/LDHB	2
hsa04070	Phosphatidylinositol signaling system	0.43864531	0.812762779	ITPR2/IP6K3	2
hsa04750	Inflammatory mediator regulation of TRP channels	0.43864531	0.812762779	ITPR2/ASIC2	2
hsa04914	Progesterone-mediated oocyte maturation	0.443961798	0.812762779	PGR/CCNA1	2
hsa04916	Melanogenesis	0.4492508	0.812762779	WNT6/TYRP1	2
hsa04933	AGE-RAGE	0.4597446	0.812762779	AGTR1/SERPINE1	2

	signaling pathway in diabetic complications	02			
hsa05142	Chagas disease (American trypanosomiasis)	0.470123428	0.823096049	SERPINE1/NOS2	2
hsa04650	Natural killer cell mediated cytotoxicity	0.495548617	0.831513781	MICA/HLA-A	2
hsa04668	TNF signaling pathway	0.495548617	0.831513781	LIF/CXCL10	2
hsa01200	Carbon metabolism	0.520198432	0.838358572	TKTL1/ALDH6A1	2
hsa04724	Glutamatergic synapse	0.525032641	0.838358572	GRM5/ITPR2	2
hsa04152	AMPK signaling pathway	0.553352085	0.846330358	CAB39L/CCNA1	2
hsa04114	Oocyte meiosis	0.557956363	0.846330358	ITPR2/PGR	2
hsa00230	Purine metabolism	0.576039198	0.857562115	ENTPD2/PDE10A	2
hsa04110	Cell cycle	0.576039198	0.857562115	GADD45A/CCNA1	2
hsa04210	Apoptosis	0.622987587	0.881084419	GADD45A/ITPR2	2
hsa04145	Phagosome	0.646877464	0.881084419	HLA-F/HLA-A	2
hsa04630	JAK-STAT signaling pathway	0.654573416	0.881084419	LIF/EPOR	2
hsa04150	mTOR signaling pathway	0.676868549	0.881514371	WNT6/CAB39L	2
hsa05164	Influenza A	0.691079871	0.881514371	HSPA2/CXCL10	2
hsa04062	Chemokine signaling pathway	0.782020436	0.916213292	CXCL10/FGR	2
hsa05206	MicroRNAs in cancer	0.837129846	0.938052536	TP63/APC	2
hsa04014	Ras signaling pathway	0.859718477	0.93944805	EFNA5/FGF5	2

Note:

Pathway ID: KEGG pathway number

Description: Function description corresponding to the KEGG pathway number

P value: *P* value of significance test

FDR-adjusted *P*-Value: *P* value of multiple hypothesis test correction

Gene ID: Differentially expressed genes in this pathway

Count: Number of differentially expressed genes in this pathway

Supplementary Table S4: The Primers of 11 fusion genes for Real time-PCR

Sample ID	Fusion Gene	Forword Primer	Reverse Primer	Fluorescence probe(5'FAM, 3'BHQ1)
S01 (Grade II)	RNF213-SLC26A11	TGAAGATGGAATCCGTAGCG	CAGAAGCACAGCAGGAAGGT	TCAGCTAAGGCTGGACCTGCTGCC
S01/ S02/ S03/ S04 (Grade II)	GATSL2-GTF2I	AACTTGCCTTCCTGTCCTCC	GTAGCTCATTGGCCTTTGGT	TCCTCCAAGACCAGATGATGATTATT
S03 (Grade II)	CPD-SEZ6	ATCACAAACGGCGCACATTG	TCCAGGATCGTGGCAGGAAG	ATGATGTGGAAGTGACTTCCTGCCA
S06 (Grade IV)	RC3H1-PKLR	CCCAGCAAACTTGAACCGACTA	TCCAACACGGAGGAAGCCAC	ATGATGTGGAAGTGACTTCCTGCCA
S06 (Grade IV)	MEX3B-KIAA1199	TACCCAGTTCTGAGCATGTCG	GCAGTGGCCCAAAGAGTTAT	TGGGGCGGCAAGATCAAGGACGTTG
S08 (Grade IV)	ABL2-NCF2	TCGCTCAGGTACGAGGGACG	TCCTTGGGTTCATCTGGAAA	CAGATGGCAAGGGTGACCAAGGCTT
S09 (Grade IV)	KIAA1549-BRAF	CGTCCACAACTCAGCCTACA	TCCATCACCACGAAATCCTT	ATCGGATGCCCAGACTTGATTAGAG
S09 (Grade IV)	CASK-SUGP1	CATTGTAGAGTTATTGGAGAC	GGGAGCAACCCCAAACCACC	ACATGGTTTTCGAATTGAAAGGCT
S09 (Grade IV)	TMEM165-PDGFRA	GTCGAGAAAGGCAAAGGCATC	TCGGGGACCGTCAAAGTGTA	TCGAGAAAGGCAAAGGCATCACA
S09 (Grade IV)	SLC35F1-ECHDC1	CTTGTGGGAAGACATCAGGG	GCTTGGTATTATGCCCATCT	AGGGAGCAGGTTAATGACTCCAGAG
S09 (Grade IV)	TCF12-SCFD2	CAATTCAGTGGATCAGGGAG	GATTCATTAGAGCTTTGAGG	ATTCAGTGGATCAGGGAGAGTCACA
